# High-Resolution Characterization of *KIR* Genes in a Large North American Cohort Reveals Novel Details of Structural and Sequence Diversity

**DOI:** 10.3389/fimmu.2021.674778

**Published:** 2021-05-07

**Authors:** Leonardo M. Amorim, Danillo G. Augusto, Neda Nemat-Gorgani, Gonzalo Montero-Martin, Wesley M. Marin, Hengameh Shams, Ravi Dandekar, Stacy Caillier, Peter Parham, Marcelo A. Fernández-Viña, Jorge R. Oksenberg, Paul J. Norman, Jill A. Hollenbach

**Affiliations:** ^1^ Programa de Pós-Graduação em Genética, Universidade Federal do Paraná, Curitiba, Brazil; ^2^ Department of Neurology, University of California, San Francisco, CA, United States; ^3^ Department of Structural Biology, Stanford University, Palo Alto, CA, United States; ^4^ Histocompatibility & Immunogenetics Laboratory, Stanford Blood Center, Palo Alto, CA, United States; ^5^ Division of Biomedical Informatics and Personalized Medicine, University of Colorado, Denver, CO, United States

**Keywords:** NK receptors, alleles, high resolution, NGS, sequencing, population

## Abstract

The *KIR (killer-cell immunoglobulin-like receptor*) region is characterized by structural variation and high sequence similarity among genes, imposing technical difficulties for analysis. We undertook the most comprehensive study to date of *KIR* genetic diversity in a large population sample, applying next-generation sequencing in 2,130 United States European-descendant individuals. Data were analyzed using our custom bioinformatics pipeline specifically designed to address technical obstacles in determining *KIR* genotypes. Precise gene copy number determination allowed us to identify a set of uncommon gene-content *KIR* haplotypes accounting for 5.2% of structural variation. In this cohort, *KIR2DL4* is the framework gene that most varies in copy number (6.5% of all individuals). We identified phased high-resolution alleles in large multi-locus insertions and also likely founder haplotypes from which they were deleted. Additionally, we observed 250 alleles at 5-digit resolution, of which 90 have frequencies ≥1%. We found sequence patterns that were consistent with the presence of novel alleles in 398 (18.7%) individuals and contextualized multiple orphan dbSNPs within the *KIR* complex. We also identified a novel KIR2DL1 variant, Pro151Arg, and demonstrated by molecular dynamics that this substitution is predicted to affect interaction with HLA-C. No previous studies have fully explored the full range of structural and sequence variation of *KIR* as we present here. We demonstrate that pairing high-throughput sequencing with state-of-art computational tools in a large cohort permits exploration of all aspects of *KIR* variation including determination of population-level haplotype diversity, improving understanding of the *KIR* system, and providing an important reference for future studies.

## Introduction

A variety of membrane-bound receptors control the response of natural killer cells (NK) to infected or malignant cells (1, 2). The killer-cell immunoglobulin-like receptors (KIR) are the most polymorphic family of NK receptors and encoded by a gene family located at chromosomal region 19q13.4 (3, 4). The *KIR* genes exhibit extraordinary variation, both within populations and between them (5–7). Although *KIR* gene-content has been extensively studied in numerous populations worldwide (8–11), less is known about *KIR* allele diversity. The unusual structural variation of the *KIR* region, coupled with the numerous alleles for each *KIR* gene (12) and extensive sequence similarity within the *KIR* gene family are distinguishing characteristics of *KIR* variation. Further characterizing the *KIR* region are frequent duplications, large deletions, hybrid genes and recombinant alleles (13–17). Together, these obstacles have impeded high-resolution allelic characterization of all *KIR* genes in population studies, which has been accomplished in only a few studies (18–21).

Transduction of NK cell activating and inhibitory signals is achieved by a subset of human leukocyte antigen (HLA) class I molecules, which serve as KIR ligands (22–24). These two interacting molecule families evolve as a unique and integrated system (25–28), and combinations of KIR and HLA have been associated with numerous diseases (29–32), including autoimmune disorders (33–36), malignancies (37–39) and infections (40–43). Combinations of KIR and HLA class I also affect placentation and the success of reproduction (44–47). Therefore, high-resolution allelic analysis of *KIR* and *HLA* class I diversity across populations will be necessary to understand their evolution and lay a foundation for functional studies to determine disease mechanisms. To facilitate this progression, we have used our custom *KIR* genotyping and bioinformatics pipeline to interrogate *KIR* diversity in a sample of 2,130 US residents. These methods were explicitly designed to cope with the complexities of *KIR* alleles, gene and haplotypes. We describe the most comprehensive analysis to date of the *KIR* genes, exploring copy number variation, haplotype patterns, and novel variation not previously reported.

## Material and Methods

### Study Population

We analyzed a cohort of 2,130 unrelated healthy adult individuals previously described by Hollenbach et al., 2019 (48). All individuals self-identified as being of European descent and were resident of the United States.

### *KIR* Genotyping

DNA samples were sequenced for all *KIR* genes, according to Norman and collaborators (49). After sequencing, raw fastq files were analyzed using our custom bioinformatics pipeline PING (Pushing Immunogenetics into the Next Generation) to obtain *KIR* gene content and allelic genotypes from next-generation sequencing (NGS) data (49). We applied an updated version of the pipeline that precisely determines the copy number of each locus through multiple alignment and filtration steps, also accurately identifying *KIR* genotypes. The updated pipeline increased the accuracy of *KIR* genotype determination and is publicly available (50).

### Haplotype Estimation

Gene-content haplotypes were identified manually, based on the precise copy number determination, the known linkage disequilibrium among *KIR* genes, and allelic information. Uncommon haplotypes were identified based on previous observations (13, 51, 52). Candidates for novel gene-content haplotypes were identified when paired with common haplotypes and observed in two or more individuals. After identifying gene-content haplotypes, we inferred the haplotypes of their *KIR* alleles using the expectation-maximization (EM) algorithm and the R package haplo.stats (http://CRAN.R-project.org/package=haplo.stats).

### Linkage Disequilibrium (LD) Analysis

Allelic genotyping data were transformed into an Arlequin entry file (.arp) using GenAlEx 6.5 (53). Gametic phase estimation using an EM algorithm and further pairwise linkage disequilibrium analysis were performed using Arlequin 3.5.2.2 (54).

### Identification of Novel Alleles

We searched for the single nucleotide variants (SNV) in *KIR2DL1* and *KIR3DL1S1* that were identified by our software but not present in any allele listed at the Immuno Polymorphism Database (IPD)-KIR release 2.9.0 (12). For individuals carrying a candidate novel SNV in *KIR2DL1* or *KIR3DL1S1*, the respective genes were re-sequenced using the Sanger method (55) using previously described primers (25, 56).

### Simulations of Molecular Dynamics

In silico, the KIR2DL1 chain was isolated from the KIR-HLA complex (PDB ID: 1IM9) (57). To map the *rs200879366* variation on the KIR2DL1 structure, the proline at position 151 was replaced by arginine using the Mutate plugin in Visual Molecular Dynamics (VMD) package (58). Both allotypes were solvated in separate simulation boxes using TIP3P solvent, and the ion concentration was adjusted to 150 KCl. Energy minimization was then performed on both systems for 150,000 steps. To mimic the anchorage of KIR2DL1 to the lipid membrane, the atom of residue 200 was fixed in space. Conformational transitions of KIR2DL1 allotypes were modeled using the NAMD software package (59) and the CHARMM36 forcefield (60) in NPT ensembles. Temperature and pressure were maintained at 310 K and 1 bar using the Langevin thermostat and Langevin piston Nose-Hoover, respectively. Periodic boundary condition in all directions and a timestep of 2fs were used. Simulations on both systems ran for 100ns. The angle between the D1 and D2 domains is the leading indicator of KIR2DL1 conformational transition. It was obtained by aligning the corresponding atom selections and calculating rotation and displacement at every timeframe using in-house tcl scripts. Structure visualizations were performed using VMD.

## Results

### *KIR* Gene Copy Number Analysis Identified Numerous Deletions and Duplications Involving *KIR3DP1*, *KIR2DL4*, and *KIR3DL1S1*

We determined *KIR* gene copy number for all 13 *KIR* genes (*KIR2DL1*, *KIR2DL23*, *KIR2DL4*, *KIR2DL5A*, *KIR2DL5B*, *KIR2DS1*, *KIR2DS2*, *KIR2DS3*, *KIR2DS4*, *KIR2DS5*, *KIR3DL1S1*, *KIR3DL2*, *KIR3DL3*) and the two pseudogenes (*KIR2DP1*, and *KIR3DP1*). Carrier frequencies and gene frequencies, based on the direct counting of all copies for each *KIR*, are given in **Supplementary Table 1**.

**Figure 1:**
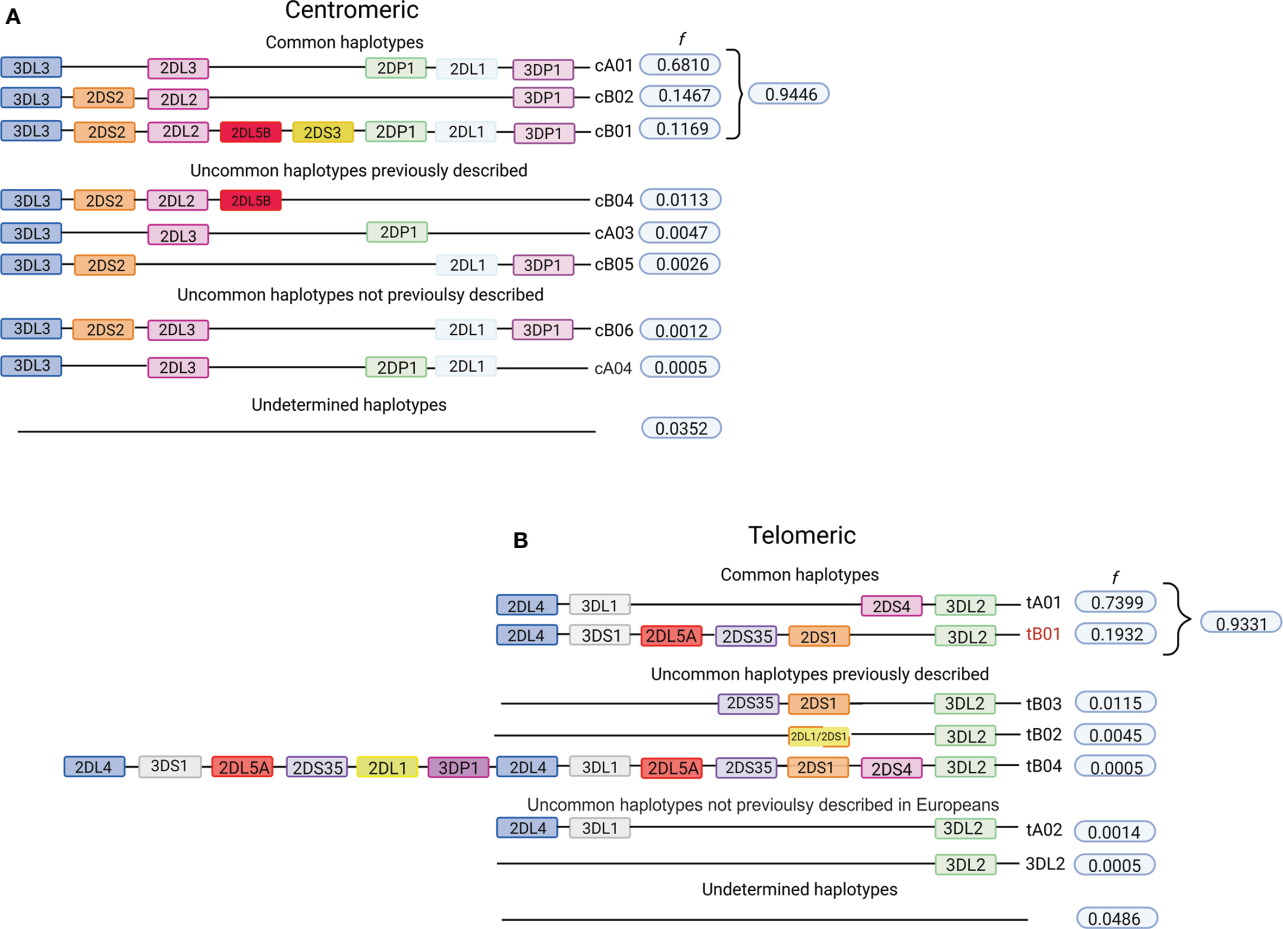
Telomeric and centromeric gene-content haplotypes in European-Americans. Although multiple variations of the *KIR* full-length haplotypes have been described, most are multiple variations of a few centromeric and telomeric haplotypes. The centromeric and telomeric regions of *KIR* haplotypes are flanked by the genes *KIR3DL3*-*KIR3DP1* and *KIR2DL4*-*KIR3DL2*, respectively, which are referred to as framework genes (62, 63). **(A)** Frequencies of centromeric *KIR* gene-content haplotypes in the study population. **(B)** Frequencies of telomeric *KIR* gene-content haplotypes in the study population. All listed haplotypes that were not previously described, we have observed in multiple individuals and in combination with a high frequency haplotype, allowing their inference with confidence. All uncommon haplotypes for which the phase could not be determined are grouped as “undetermined”. Figure created with BioRender.com.

The *KIR A* haplotype is defined by the presence of only one gene encoding a short-tailed activating receptor (KIR2DS4) and a fixed number of genes encoding inhibitory KIR. In contrast, *KIR B* haplotypes encode various combinations of activating and inhibiting receptors (61). A total of 31.4% of individuals in the cohort of 2,130 Europeans are homozygous for the full-length *KIR A* haplotype (*cA01*~*tA01*/*cA01*~*tA01*, *f* = 0.58). The centromeric and telomeric portions of the *KIR* haplotype are flanked by the framework genes *KIR3DL3-KIR3DP1* and *KIR2DL4-KIR3DL2*, respectively (62, 63). On analyzing these two regions separately, we observed that 94% of the centromeric diversity is explained by just three gene-content haplotypes (*cA01*, *cB02*, and *cB01*; **Figure 1A**), whereas *tA01* and *tB01* correspond to 93% of the telomeric haplotypes (**Figure 1B**). We also indentified the presence of two novel centromeric haplotypes, and two telomeric haplotypes not previoulsy described in Europeans. The novel *cB06* haplotype differs from *cB01* by lacking *KIR2DP1*, and *cA03* differs from the more common *cA01* by lacking *KIR3DP1*. Present in the telomeric region is *tA02*, which only differs from *tA01* by lacking *KIR2DS4*. Of particular interest is a haplotype observed in two individuals that has only the *KIR3DL2* framework gene in the telomeric region. The gene content and organization of 3.5% of the centromeric and 4.9% of the telomeric haplotypes could not be determined.

**Figure 2:**
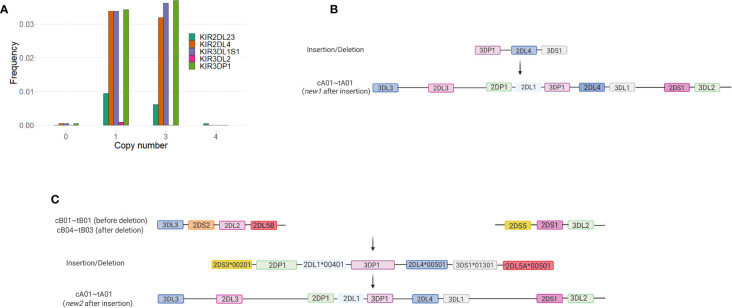
Deletions and duplications involving *KIR2DL4*, *KIR3DL1S1*, and *KIR3DP1* were observed in more than 6% of *KIR* haplotypes Together with *KIR2DL23* and *KIR3DL1S1*, framework genes (*KIR3DL3*, *KIR3DP1*, *KIR2DL4*, and *KIR3DL2*) were initially considered to be present in all *KIR* haplotypes, with only rare exceptions. Here, we show that deletions and duplications in these genes are relatively frequent in the study population. **(A)** Frequencies of gene copy number for individual genes. **(B)** Suggested origin of the novel haplotype containing a duplication of *KIR3DP1~KIR2DL4~KIR3DS1* and observed in 38 individuals (*f* = 0.009). Created with BioRender.com. **(C)** Suggested origin of the novel haplotype containing a duplication of *KIR2DS3~KIR2DP1~KIR2DL1~KIR3DP1~KIR2DL4~KIR3DS1~KIR2DL5A*, observed in 19 individuals (*f* = 0.004). Figure created with BioRender.com.

Of the framework genes, *KIR2DL4* has the most copy number variation in our study. We observed 72 individuals (3.4%) carrying one copy of *KIR2DL4* and 67 (3.1%) carrying three copies (**Figure 2A**). In this cohort, deletion of *KIR2DL4* is invariably accompanied by the deletion of *KIR3DL1S1*. *KIR3DP1* is deleted in 63 of the 72 (87.5%) haplotypes having the *KIR2DL4*~*KIR3DL1S1* deletion. *KIR3DL1S1* and *KIR3DP1* are duplicated in 63 out of 68 individuals carrying duplications of *KIR2DL4*. Insertion of *KIR3DP1~KIR2DL4~KIR3DS1* into a *tA01* haplotype created a novel haplotype, carried by 38 individuals (*f* = 0.009; **Figure 2B**). Insertion of the segment KIR2DS3*00103~KIR2DP1~KIR2DL1*00401~KIR3DP1~ KIR2DL4*00501~KIR3DS1*01301~KIR2DL5A*00501 also into *tA01* gave another novel haplotype observed in 19 individuals (*f* = 0.004; **Figure 2C**). In addition, we always observed the centromeric *cB04* haplotype to be in the same gametic phase as *tB03*, and *cA03* always with *tB02*. Duplication of *KIR2DL2* present in *cB02* was observed in 7 individuals (*f* = 0.002)

### Several *KIR* Haplotypes Are Marked by Specific *KIR* Alleles

**Figure 3:**
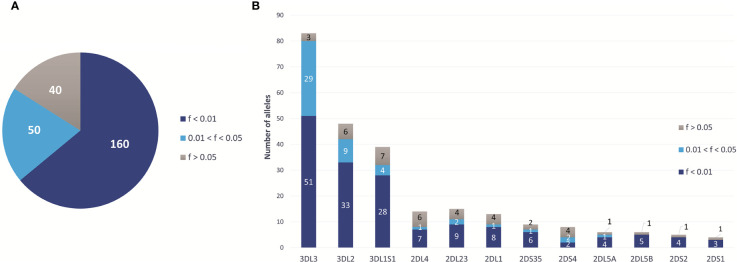
Overview of the *KIR* allelic diversity in European-Americans. **(A)** Total number of *KIR* alleles at 5-digit resolution observed in 2,130 European Americans and stratified by frequency. **(B)** Number of alleles observed for each gene (5-digit resolution), stratified by frequency.

**Figure 4:**
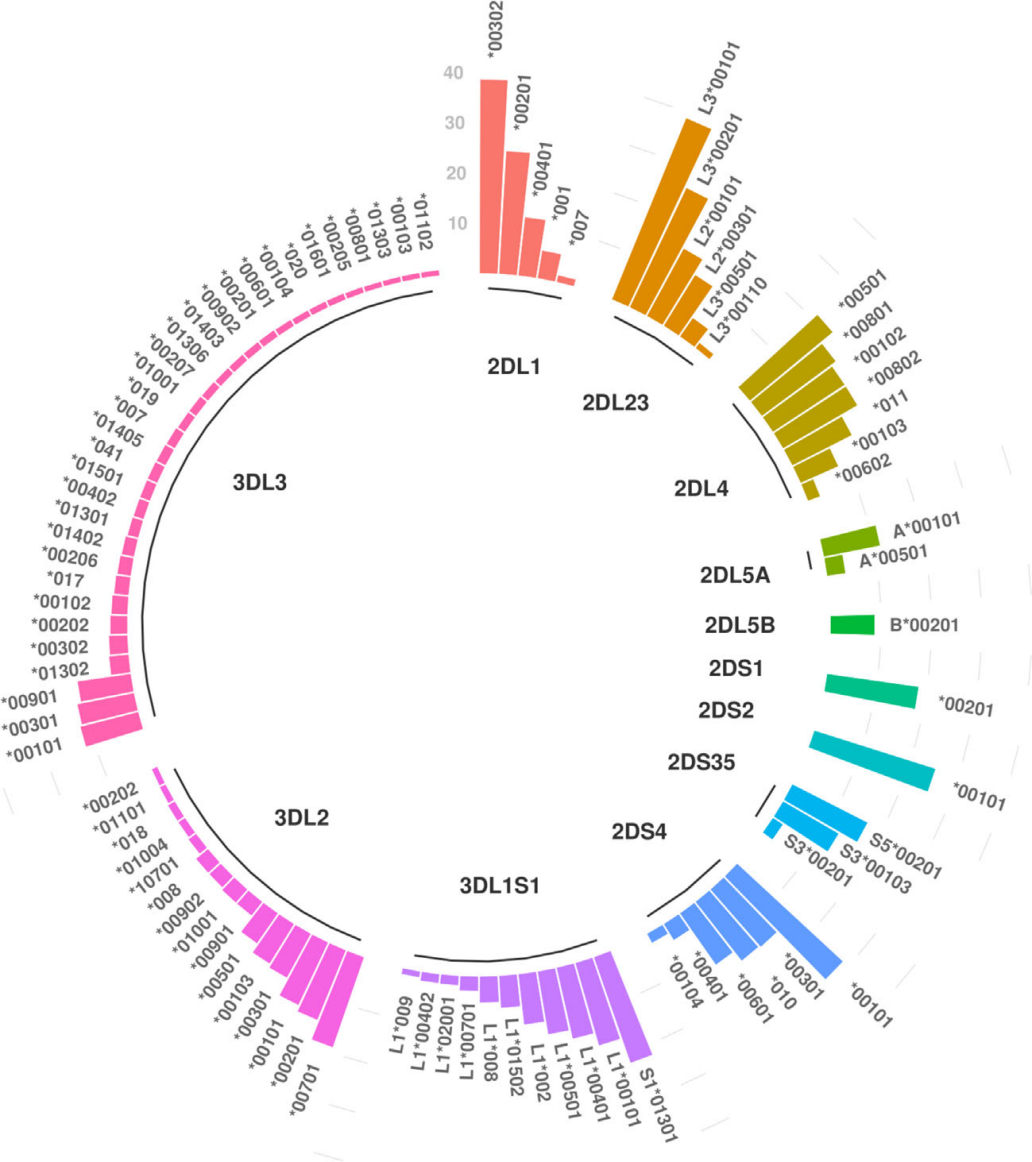
Overview of the most common alleles in European-Americans. Only alleles with frequencies greater than 1% are shown. For full list of allelic frequencies, see **Supplementary Table 2**.

**Figure 5:**
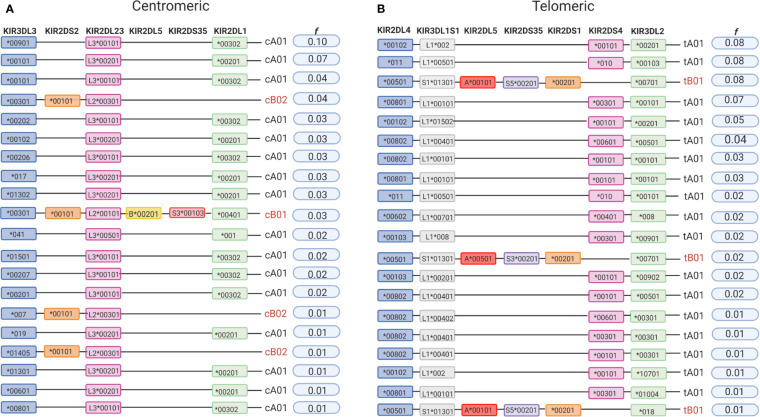
High-resolution allelic haplotypes in European-Americans. **(A)** The 20 most common centromeric *KIR* haplotypes. **(B)** The 20 most common telomeric *KIR* haplotypes observed in European Americans (n = 2,130). Created with BioRender.com.

All 13 *KIR* genes were genotyped to five-digit allele resolution in the study sample. We identified 250 *KIR* alleles, of which 90 (37.6%) have frequencies equal or greater than 0.01, and 40 (17%) have frequencies equal or greater than 0.05 (**Figure 3A**). *KIR3DL3* has the highest variety of alleles (n = 83), followed by *KIR3DL2* (n = 48) and *KIR3DL1S1* (n = 39) (**Figure 3B**). **Figure 4** summarizes *KIR* allele diversity of the cohort and complete allele frequencies are given in **Supplementary Table 2**. Among the 20 most common centromeric haplotypes, 16 are *cA01* (**Figure 5A**). Similarly, 17 of the 20 most common telomeric haplotypes were *tA01* (**Figure 5B**).

**Figure 6:**
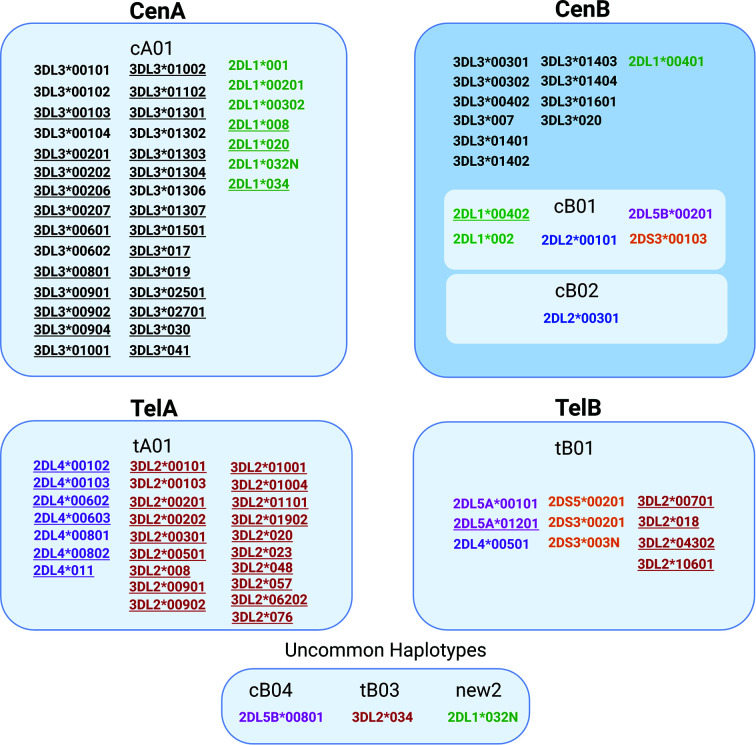
Some alleles are associated with specific *KIR* haplotypes. Alleles associated with specific haplotypes are listed inside each box. Underline marks alleles exclusively present in the specific haplotype. The other alleles were differentially associated with particular haplotypes, but not exclusively (>95% of the observations). For haplotype new2, please see **Figure 2**.

This is the first study to describe high-resolution (5-digit) *KIR* haplotypes for all functional *KIR* genes in a large population sample. Analyzing this large number of individuals gave us sufficient power to fully explore the patterns of LD and identify alleles that are exclusively or predominately associated with specific haplotypes (**Figure 6**). For example, the alleles KIR3DL2*00701 and *018 were observed solely in *tB01* haplotypes, whereas KIR3DL2*00103, *00201, *00501, *00901, *00101, and *008 were observed only in *tA01*. Similarly, KIR3DL3*00301 and *00402 are characteristic of *CenB*, whereas *00901 and *00101 are exclusive to *CenA* haplotypes. With few exceptions, 2DL4*00501 is the only *KIR2DL4* allele found in *tB01* haplotypes (99.4%), being in complete LD with KIR3DS1*013. Additionally, a few low-frequency alleles are associated with specific, uncommon haplotypes; for example, KIR3DL2*034 (*f* = 0.002) is present only in *tB03*, and KIR2DL5B*00801 (*f* = 0.004), is present only in *cB04* (*f* = 0.01). Furthermore, for the first time we describe multiple high-resolution allelic configurations of the full-length *cB04~tB03* haplotype (**Supplementary Table 3**).

**Figure 7:**
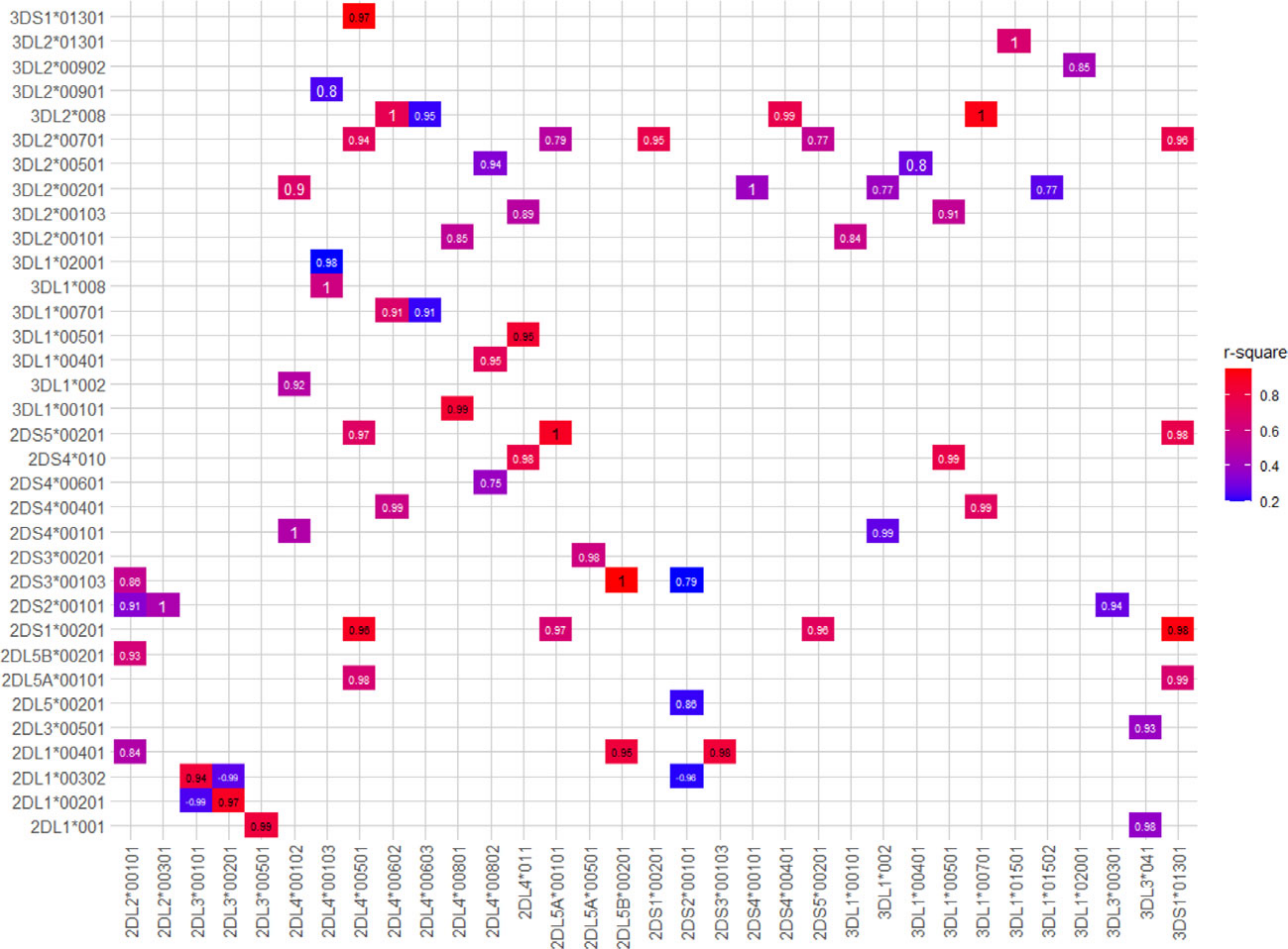
Strong linkage disequilibrium (LD) between *KIR* alleles. Boxes represent the pairs of *KIR* alleles with the strongest LD in the present study. D’ values are written inside of each box and color scale represent r^2^ values. Only pairs with r^2^ > 0.2, D’ > 0.7 were shown. The *p*-value of all pairs was < 10^-5^.

As well as the haplotypic associations, we observed many instances of strong LD among specific sets of *KIR* alleles (**Figure 7**). In summary, KIR2DL4*00501, KIR3DS1*01301, KIR2DS1*00201, KIR2DL5A*00101, KIR2DS5*00201, and KIR3DL2*00701 are frequently observed together. Many other *KIR2DL4* alleles are in strong LD with specific *KIR3DL1* alleles. Specific examples are KIR2DL4*00801 with KIR3DL1*00101 (D’ = 0.99, r^2^ = 0.85); KIR2DL4*011 with KIR3DL1*00501 (D’ = 0.95, r^2^ = 0.85); *KIR*2DL4*00802 with KIR3DL1*00401 (D’= 0.95, r^2^ = 0.73); KIR2DL4*00602 with KIR3DL1*00701 (D’ = 0.91, r^2^ = 0.68); and KIR2DL4*00103 with KIR3DL1*008 (D’ = 1, r^2^ = 0.6). In the centromeric region, KIR2DL5B*00201 and KIR2DS3*00103 are always observed together. KIR2DL1*00401 is in strong LD with KIR2DS3*00103 (D’ = 0.98 and r^2^ = 0.83) and KIR2DL5B*00201 (D’ = 0.95 and r^2^ = 0.82), whereas KIR2DL3*00201 is associated with KIR2DL1*00201 (D’ = 0.97, r2 = 0.89). A full list of LD values for pairs of *KIR* alleles is given in **Supplementary Table 4**.

### Numerous Novel *KIR* Variants Are Present in the Cohort of European Americans

In this analysis of 2,130 individuals, we identified 398 individuals (18.7%) carrying at least one *KIR* recombinant allele that do not correspond to any sequences deposited in the *KIR* database. We define as recombinant allele those that are characterized by different phasing combinations of previously known variable sites. These observations are likely to represent the presence of new alleles that were not present or not detected in previous studies of *KIR* variation. *KIR3DL1S1* accounts for 33% of the observations of candidate new alleles, corresponding to a total allelic frequency of 0.04 at this locus. A large proportion of individuals carrying possible novel alleles were also observed for other KIR2D and KIR3D genes (**Table 1**).

**Table 1:** Large proportion of individuals carrying possible novel alleles.

Locus	n	*f*
*KIR3DL1S1*	154	7.23%
*KIR3DL2*	57	2.68%
*KIR2DL5*	56	2.63%
*KIR2DL4*	53	2.49%
*KIR3DL3*	48	2.25%
*KIR2DL1*	43	2.02%
*KIR2DL23*	31	1.46%
*KIR2DS3*	24	1.13%
*KIR2DS5*	10	0.47%
*KIR2DS4*	5	0.23%

n, absolute number individuals carrying potential at least one possible novel allele; f, relative frequency of individuals carrying possible novel alleles.

In addition to these candidate novel *KIR* recombinant alleles, we also used our software to identify possible novel SNVs. In some cases, these SNVs may have been reported in the dbSNP database (64), but they were not associated with any *KIR* allele sequence deposited in the IPD-*KIR* database release 2.9.0 (12). Therefore, in the context of *KIR*, these SNVs would be contributing to novel alleles that each differ from a known *KIR* allele by a single nucleotide substitution. To confirm the sequences of novel variants, we used the Sanger method to re-sequence individuals carrying any possible novel SNV in *KIR2DL1* and *KIR3DL1S1*. While confirmation of all novel variants was out of scope for the current project, we selected these loci as exemplars for this work due to substantial previous work examining their structure and function (25, 56, 61, 65–71), including the availability of crystal for molecular modeling structures (57, 72–76). In future work we will continue to explore novel variants that were detected at other loci during this study.

For *KIR2DL1*, 8 of 30 variants were confirmed by Sanger sequencing, while 10 of 32 variants were similarly confirmed for *KIR3DL1S1*. Most of the SNVs in *KIR3DL1S1* were observed in only a single individual, except for two synonymous variants, *rs754894112* and *rs1462310393* (**Table 2**). In contrast, the majority of confirmed novel variants in *KIR2DL1* were observed in several individuals (**Table 3**). Interestingly, 14 out of the 18 confirmed variants in these two genes were non-synonymous substitutions, with functional effects ranging from conservative to radical according to the Grantham scale of physicochemical distances between amino acids (77).

**Table 2:** Ten confirmed novel single nucleotide variants in *KIR3DL1S1*.

rsID	n	Exon	Change	Mature protein	Amino acid	Grantham
rs771871523	1	3	G>T	12	Ala>Ser	99
rs1272096635	1	4	C>A	107	Ala>Asp	126
rs769147743	1	4	T>C	127	Ile>Thr	89
rs200893904	1	4	C>T	171	Thr>Ile	89
rs1182774591	1	4	G>C	187	Ala>Pro	27
new SNP	1	5	G>C	249	Arg>Pro	103
rs754894112	12	5	C>T	266	synonymous	
rs1462310393	2	5	C>A	275	synonymous	
new SNP	1	7	C>T	319	His>Tyr	83
rs984592565	1	9	G>A	392	Arg>His	29

These variants have been have been reported and assigned rsID in the dbSNP database (64), but not present in any KIR allele deposited in the IPD-KIR database release 2.9.0 (12), therefore representing novel KIR alleles differing from others by only one nucleotide position. New SNP represent variants not previously assigned rsID in the dbSNP database.

**Table 3:** Eight confirmed novel single nucleotide variants in *KIR2DL1*.

rsID	n	Exon	Change	Mature protein	Amino acid	Grantham
rs201225013	7	1	A>G	signal peptide	Met>Val	21
rs148427642	4	1	T>G	signal peptide	Cys>Gly	159
rs749640662	13	5	G>A	120	Ala>Thr	58
rs200879366	8	5	C>G	151	Pro>Arg	103
rs749653872	3	5	C>T	157	synonymous	
rs570412759	16	7	G>A	245	Arg>Hist	29
rs201527316	1	9	G>A	296	Arg>Hist	29
rs778821930	2	9	C>A	309	synonymous	

These variants have been have been reported and assigned rsID in the dbSNP database (64), but not present in any KIR allele deposited in the IPD-KIR database release 2.9.0 (12), therefore representing novel KIR alleles differing from others by only one nucleotide position.

### Simulation of Molecular Dynamics Predicts That Dimorphism in Codon 151 of *KIR2DL1* Affects Binding to HLA Class I

**Figure 8:**
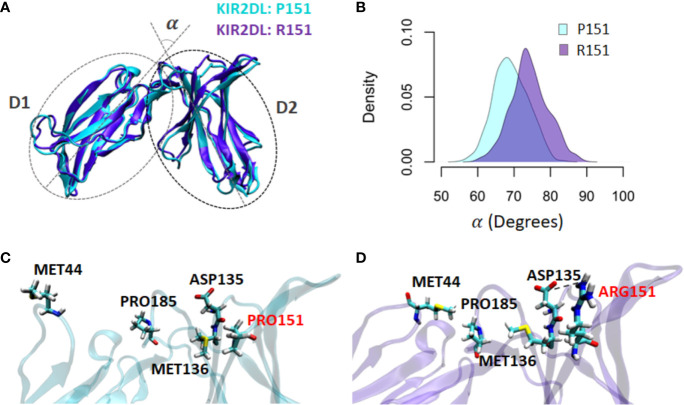
Pro151Arg substitution in KIR2DL1 is predicted to affect the stability of its binding to HLA-C. The impact of the Pro151Arg mutation on the KIR2DL1 conformation and its HLA-C binding site was studied by simulating both the mutant and wildtype structures**. (A)**. The angle between D1 and D2 domains (alpha) of KIR2DL1 was computed in both allotypes as an indicator of conformational transition from the HLA-bound to a free state. **(B)** The alpha angle in the mutant is closer to the HLA-bound state, whereas the transition to the unbound state occurs smoothly in the wildtype. **(C)** The interaction between Met44 and Pro185 was disrupted upon angle change between D1 and D2 in the wildtype. **(D)** Arg151 forms an interaction with Asp135, which also leads to new interactions between Met136, Pro185, and Met44, maintaining the open conformation of the mutant.

To explore the functional differences of *KIR2DL1* alleles that differ by a single nucleotide, we simulated and compared the molecular dynamics simulations of KIR2DL1 allotypes that differ by the non-conservative substitution of proline to arginine at position 151. Underlying this difference is rs200879366*C>*G*. Different conformations were sampled during the simulation trajectory, so that each time step features an individual conformer. The angle change mediated the transition between free and HLA-bound states of KIR2DL1 between the Ig domains (D1 and D2) that eventually affected the HLA binding region (**Figure 8A**). Within 100ns of simulation, the angle between the D1 and D2 domains was decreased by 10° in the wildtype (Pro) but was not perturbed in the mutant (Arg) (**Figure 8B**). The conformational transition appears to be mediated by a network of interactions spanning from Met44 to Arg 151. In the wildtype, Met44 is released from Pro185, allowing the angle between D1 and D2 to decrease (**Figure 8C**). By contrast, the angle is increased in the mutant. This enables Arg151 to form a salt bridge with Asp135, leading to an interaction between Met136 and Pro185 (**Figure 8D**) that allows Pro185 to more strongly associate with Met44. This set of localized rearrangements in KIR2DL1 is likely essential for its stable binding to HLA class I.

## Discussion

The general configuration of *KIR* gene-content haplotypes was first described two decades ago, when it was observed that four framework genes separate two distinct sub-clusters of genes (7, 24, 62, 78, 79). *KIR3DL3* and *KIR3DP1* were seen to delimit the centromeric region, while *KIR2DL4* and *KIR3DL2* delimit the telomeric region of the *KIR* gene family. The other two genes that are present in most *KIR* haplotypes are *KIR2DL23* and *KIR3DL1S1* (6). Although deletions and duplications of these genes have been previously reported (15–17, 80–82), technical limitations have precluded direct copy number determination of all *KIR* in large-scale population studies. We show here that large structural deletions and duplications involving the framework genes are relatively frequent in European-descendant individuals. For instance, more than 6% of individuals carry a deletion or duplication of *KIR2DL4*. Similar to haplotype variants described in the literature (13, 51, 52), all gene-content *KIR* haplotypes lacking *KIR2DL4* also lacked *KIR3DL1S1* (*tB02* and *tB03*). These haplotypes have been described for other European-Americans (52), while an extensive study of Europeans from Germany did not seek to analyze novel gene-content haplotypes (83). Observation of *KIR2DL4*~*KIR3DL1S1* deletions at high-frequencies in Africans (18, 25) raises the possibility that these variant *KIR* haplotypes originated prior to the modern human migration out of Africa, and that they might also be present in most worldwide populations.

Interestingly, haplotypes carrying *KIR2DL4* duplications also have duplications of *KIR3DL1S1* and *KIR3DP1.* Based on our observations, including the fact that *cB04*~*tB03* are always in phase, we propose that a single deletion of KIR2DS3*00103~KIR2DP1~KIR2DL1*00401~ KIR3DP1~KIR2DL4*00501~KIR3DS1*01301~KIR2DL5A*00501 from the haplotype *cB01~tB01* originated the *cB04~tB03* haplotype. This seven-locus fragment was possibly inserted into *cA01~tA01*, which would explain the novel full-length haplotype that we identified in multiple individuals. The large cohort that we analyzed allowed us to phase the alleles of the seven-locus indel at high-resolution, providing a unique opportunity to infer the origin of these haplotypic variants.

Previous studies described *KIR* haplotypes at lower genotyping resolution and in smaller sample sizes. For example, Vierra-Green et al. (52) described *KIR* haplotypes at 3-digit resolution in 506 Euro-Americans while Hou et al. (20) analyzed most *KIR* genes at higher resolution but in a small cohort. Here, we present the first study to show 5-digit allelic haplotypes of all *KIR* genes for a large sample of the European-descent U.S population. Notably, the most common centromeric haplotype in our study cohort, KIR3DL3*00901~ KIR2DL3*00101~KIR2DL1*00302 (*f* = 0.10), is also the most common in four African populations (Datoonga, *f* = 0.11; Baka, *f* = 0.15; Dogon, *f* = 0.18; and Fulani, *f* = 0.14) (18). This haplotype is likely the same reported as the most frequent (*f* = 0.23) in a smaller European American cohort that was not analyzed for all *KIR* genes at high resolution (20). Interestingly, two of the low-frequency telomeric haplotypes present in our sample (*tA02* and *KIR3DL2*) have not been reported in other European populations, but were observed in African populations from Mali (*3DL2*, *f* = 0.01), Democratic Republic of Congo (*tA02*, *f* = 0.08) and Tanzania (*3DL2*, *f* = 0.01; *tA02*, *f* = 0.01) (18). A limitation of determining haplotypes without family segregation studies or confirmation by long-range sequencing is the impossibility to identify unknown haplotype structures or to precisely infer those haplotypes observed in lower frequencies. For this reason, we were not able to confidently identify the less common haplotypes, therefore, presenting data only for the most common ones. However, our large population sample coupled with the curated high-quality data allow us to identify the haplotypic diversity that represent most of the *KIR* diversity in Europeans.

Our well-powered analysis of LD across the *KIR* region shows that some alleles are clearly associated with specific structural haplotypes. Because KIR2DL4*00501 and KIR3DL2*00701 are present in *tB01* and are associated with other *tB01-*associated alleles, such as KIR3DS1*01301, it was possible to verify that the insertion of fragments containing *KIR3DS1* occurred on the *cA01~tA01* haplotype. In some cases, specific alleles also associate with uncommon haplotypes, which may be used as markers for these unique haplotypes. These examples highlight how our detailed LD information for high-resolution *KIR* sequencing constitutes a significant resource, yielding valuable information that will facilitate the comprehension and identification of *KIR* haplotypes in future studies.

With the development of robust high-resolution *KIR* typing methods, the discovery of novel *KIR* variants has become more achievable. However, short read misalignment is a major confounding factor in discovering novel variants, particularly those differing from known variants by only one nucleotide. Because the identification of possible novel SNVs is overall not likely in comparison to the possibility of being artifacts due to misalignment of highly similar sequence reads, our pipeline is initially set to exclude them from the primary analysis but flag them for further detailed inspection. This is a limitation intrinsic to short-sequence data analysis in the context of the *KIR* sequence homology, and rare novel variants might be missed. Aiming for an overview of the extent of this methodological limitation, we applied Sanger method to re-sequence all possible novel variants in two highly polymorphic genes, regardless of their frequencies. Although we confirmed approximately one-third of the possible novel SNVs for *KIR3DL1S1* and *KIR2DL1*, most of these variants were observed in only one or a few individuals. In other words, even though our pipeline can possibly miss some of the novel variation, the new SNVs do not represent significant overall distortions in our dataset. In fact, our method has been proven to show an overall high performance for determining accurate genotypes (median 96.5%), and only 1% to 3% of unresolved genotypes (50).

Low frequent variants may, however, be of particular interest especially if they may cause impact on the receptors’ function. It is remarkable that most confirmed novel variants cause moderate to radical non-synonymous substitutions, ultimately leading to functional protein variation. To provide new insights to the functional significance of describing novel variants, we focused on variant rs200879366*G, which was previously reported with a frequency of 0.01 in the Finnish population (84) but had not previously been associated with a specific *KIR* allele. Although residue 151 is in the D2 domain it does not make direct contact with the HLA-C ligand. Nevertheless, polymorphism at the neighboring residue, position 154, has been implicated in differential avidity for the HLA-C ligand (85). Our prediction shows that Asp135, which is directly engaged in HLA binding, forms a bond with Arg151, allowing us to speculate that rs200879366*G may result in reduced binding to HLA. This example demonstrates the likelihood that many other functional variants will be identified as we interrogate *KIR* allelic diversity in worldwide populations.

According to the recently updated IPD-KIR database (Release 2.10.0, 16 December 2020), the variant rs200879366*G marks two unconfirmed alleles, KIR2DL1*044 and KIR2DL1*046, corroborating our findings. These unconfirmed allele sequences were freshly submitted by the same group that genotyped *KIR* in over a million European samples from the DKMS donor registry (86). Although a remarkable effort in its scale and importance to the field, that study targeted specific exons of each *KIR*, resulting in a 3-digit resolution genotyping with substantial ambiguities. In contrast, our study sought to analyze all *KIR* exons and introns of each gene (5-digit resolution). While smaller than the DKMS study by orders of magnitude, our study is nevertheless the largest sample to-date to comprehensively analyze all aspects of *KIR* variation at this resolution, including copy number, allele-haplotype associations, pairwise LD, and functional consequences of novel variation.

For decades, most *KIR* studies in populations were limited to analyzing the presence and absence of genes (5, 7, 9, 62, 87–89). The study of *KIR* gene content laid the basis of the field and suggested that *KIR* diversity and plasticity were and may still be ahead of our technical capabilities. Here, we aimed to set new ground for exploring *KIR* diversity by providing the first large-scale study to deeply analyze copy number variation and high-resolution allelic variation of all genes in a large population sample from the United States. Our results show a large proportion of multi-locus deletions and duplications of genes that were until recently considered rare, in addition to unusual gene-content haplotypes and a high frequency of novel alleles. We argue that as we continue to interrogate *KIR* at high-resolution, we will continue to uncover more layers of this region’s complexity, discovering frequent novel variants with functional relevance that have been previously missed due to technical limitations.

## Data Availability Statement

The original contributions presented in the study are included in the article/**Supplementary Material**. Further inquiries can be directed to the corresponding author.

## Author Contributions

JH, PN, and DA designed the study. NN-G, GM-M, and DA performed NGS and Sanger sequencing. LA and DA analyzed the data. WM and RD contributed with bioinformatics analysis. HS performed molecular dynamics simulations. SC managed and organized the samples. PP, MF-V, JO, PN, and JH contributed with samples and/or reagents. LA, DA, and JH drafted the manuscript. All authors contributed to the article and approved the submitted version.

## Funding

This work was supported by grants U19NS095774 and U01 AI090905 from the U.S. National Institutes of Health (NIH). The UCSF DNA biorepository is supported by Si-2001-35701 from the National Multiple Sclerosis Society.

## Conflict of Interest

The authors declare that the research was conducted in the absence of any commercial or financial relationships that could be construed as a potential conflict of interest.

## Supplementary Material

Supplementary Table 1
*KIR* gene-content frequencies in European-Americans.

Supplementary Table 2 High-resolution allelic frequencies of the *KIR* genes in European-Americans.

Supplementary Table 3 Multiple configurations of the haplotype *cB04~tB03* in European-Americans.

Supplementary Table 4 Pairwise linkage disequilibrium between *KIR* alleles. Only pairs with r2 > 0.2 and D’ > 0.7 are shown.
